# Comparative Analysis of Phenolic Profiles and Antioxidant Activity in the Leaves of Invasive *Amelanchier × spicata* (Lam.) K. Koch in Lithuania

**DOI:** 10.3390/plants14020221

**Published:** 2025-01-14

**Authors:** Sandra Saunoriūtė, Kristina Zymonė, Mindaugas Marksa, Lina Raudonė

**Affiliations:** 1 Department of Pharmacognosy, Faculty of Pharmacy, Lithuanian University of Health Sciences, Sukileliu Av. 13, LT-50162 Kaunas, Lithuania; lina.raudone@lsmu.lt; 2 Botanical Garden, Vytautas Magnus University, Z. E. Zilibero Str. 4, LT-46324 Kaunas, Lithuania; 3 Laboratory of Biopharmaceutical Research, Institute of Pharmaceutical Technologies, Lithuanian University of Health Sciences, Sukileliu Av. 13, LT-50162 Kaunas, Lithuania; kristina.zymone@lsmu.lt; 4 Department of Analytical and Toxicological Chemistry, Faculty of Pharmacy, Lithuanian University of Health Sciences, Sukileliu Av. 13, LT-50162 Kaunas, Lithuania; mindaugas.marksa@lsmu.lt

**Keywords:** *Amelanchier × spicata*, dwarf serviceberry, invasive plant, phenolics, antioxidant activity

## Abstract

The environmental impact of invasive species necessitates creating a strategy for managing their spread by utilising them as a source of potentially high-value raw materials. *Amelanchier × spicata* (Lam.) K. Koch (dwarf serviceberry) is a shrub species in the *Rosaceae* Juss. family. The evaluation of different populations of plants that accumulate great amounts of biologically active compounds is requisite for the quality determination of plant materials and medicinal and nutritional products. The assessment of natural resources from a phytogeographic point of view is relevant. Phytochemical analysis of *A. spicata* leaf samples was carried out using spectrophotometric methods, HPLC-PDA, and HPLC-MS techniques, while antioxidant activity was determined using ABTS, FRAP, and CUPRAC assays. A significant diversification of phenolic compounds and antioxidant activity was determined in the *A. spicata* leaf samples collected in different habitats. Due to their characteristic chemical heterogeneity, natural habitats lead to the diversity of indicators characterising the quality of plant raw materials. Chlorogenic acid and neochlorogenic acid, as well as quercitrin, rutin, and hyperoside, were found to be predominant among the phenolic compounds. Thus, these compounds can be considered phytochemical markers, characteristic of the *A. spicata* leaf material from northern Europe.

## 1. Introduction

*Amelanchier* Medik. is a small genus within the *Rosaceae* Juss. family, comprising around 28 recognised species [1,2]. *Amelanchier × spicata* (Lam.) K. Koch is a hybrid of *Amelanchier alnifolia* (Nutt.) Nutt. ex M. Roem. × *Amelanchier humilis* Wiegand, originating from Canada and the northern regions of the United States. It has been naturalised in various areas, including Africa, Europe, and the eastern part of Asia [3]. *A. alnifolia* (Nutt.) Nutt. ex M. Roem. (Saskatoon berry), *A. lamarckii* F.G. Schroed. (juneberry), *A. arborea* (F.Michx.) Fernald (Downy serviceberry), *A. canadensis* (L.) Medik. (Canadian serviceberry), *A. ovalis* Medik. (European juneberry), and *A. spicata* (Lam.) K. Koch (dwarf serviceberry) are the main species of the genus, receiving increased scientific attention due to their ecological importance and economic value in agriculture [4,5,6,7,8]. However, research data on the chemical composition of *A. spicata* and *A. humilis* leaves and fruits are extremely scarce. *Amelanchier* species are known for their ability to thrive in various climates [1]. The dwarf serviceberry, a North American-origin hybrid species naturalised in Europe in the 18th century, falls into the alien species group in Lithuania. The European *A. spicata* exhibits morphological differences compared to the American species, which could be attributed to various ecological, genetic, and environmental factors. The dwarf serviceberry, as an invasive species, spreads very quickly in certain habitats, such as the western, eastern, and southern parts of Lithuania. Therefore, in 2012, the Invasive Species Council under the Ministry of Environment of the Republic of Lithuania included it in the list of invasive species [9,10].

Utilising invasive plants supports sustainable development by converting ecological challenges into economically valuable resources, corresponding to the circular economy principles. Instead of only controlling invasive species through eradication, targeted utilisation of plant materials as resources of valuable secondary metabolites supports biodiversity and shifts the process towards added-value products and no waste, ultimately advancing environmental and economic resilience [11,12,13,14]. However, the phenolic profiles of *A. spicata* leaves, especially in European invasive populations, still need to be elucidated. This gap in research provides an opportunity to explore the potential uses of *A. spicata* as a natural resource towards sustainable applications and waste reduction. While the plant is considered invasive in Lithuania, it offers a sustainable source for high-value compounds that can be extracted and utilised.

As shown in various studies, fruits of the *Amelanchier* genus are rich in various biochemical compounds, with anthocyanins, flavonols, flavones, and phenolic acids as the major components [15,16,17]. Anthocyanins, including cyanidin-3-galactoside, cyanidin-3-glucoside, cyanidin-3-arabinoside, and cyanidin-3-xyloside, have been identified as abundant constituents in the fruits of these plants [8,18,19]. Phenolic acids, such as chlorogenic acid, neochlorogenic acid, cryptochlorogenic acid, gallic acid, p-hydroxybenzoic acid, and caffeic acid derivatives, are also prevalent. Carotenoids (zeaxanthin, all-*trans*-lutein, 13-*cis*-lutein, β-carotene) contribute to the chemical complexity of these plants. *Amelanchier* fruits accumulate important minerals and vitamins, including calcium, magnesium, potassium, iron, and zinc, alongside essential vitamins such as C, A, and several B vitamins. Furthermore, triterpenic compounds (betulinic, oleanolic, and ursolic acids) were determined to be present in these plants [8,20].

*Amelanchier* fruits are used in traditional medicine to treat stomach, eye, and ear diseases. The boiled bark is used as a disinfectant, and a root infusion is employed to prevent miscarriage [20]. Most *Amelanchier* species and cultivars produce fruits with potent antioxidant properties, and studies report various pharmacological effects, including anti-inflammatory, antibacterial, anti-allergic, anti-diabetic, anti-cancer, anti-atherosclerotic, and antitumor activities [2,4,5,21,22]. These properties suggest *Amelanchier* fruits have high potential for use in the food, pharmaceutical, and cosmetics industries [2,23,24,25]. To our knowledge, no data exist on the phytochemical composition of *A. spicata* leaves. Therefore, further research to elucidate the phytochemical traits of *A. spicata* leaves could provide valuable insights into the species’ chemophenetic characteristics and biomedical potential.

The aim of this study was to determine the composition of phenolic compounds and the antioxidant activity in leaf extracts of *A. spicata* and to identify habitats with distinct phytochemical compositions. The results of our research provide new, detailed knowledge about the variation in the qualitative and quantitative composition of phenolic compounds, as well as the antiradical and reductive activities, in vitro, of extracts from dwarf serviceberry leaf samples collected from different habitats in Lithuania. The composition of *A. spicata* leaf samples from the most studied species has not been investigated so far, so the phytochemical traits of *A. spicata* leaves could provide invaluable knowledge regarding the species’ chemophenetic characterisation and biomedical potential.

This study focuses on characterising the phenolic profiles of *A. spicata* leaves, investigating their antioxidant properties and the variability in phytochemical composition across distinct habitats. By identifying the phytochemical diversity in *A. spicata* leaves from different Lithuanian habitats, this research contributes critical insights into the species’ chemophenetic profile. The results are critically important to the waste reduction strategy and to inducing sustainable management practices for valuable components of invasive species. Thus, our study provides the first comprehensive assessment of the phenolic profile of *A. spicata* leaves, expanding the possibility of using invasive plant resources.

## 2. Results

### 2.1. Determination of Total Phenolic Compounds and Proanthocyanidin Content

**Figure 1:**
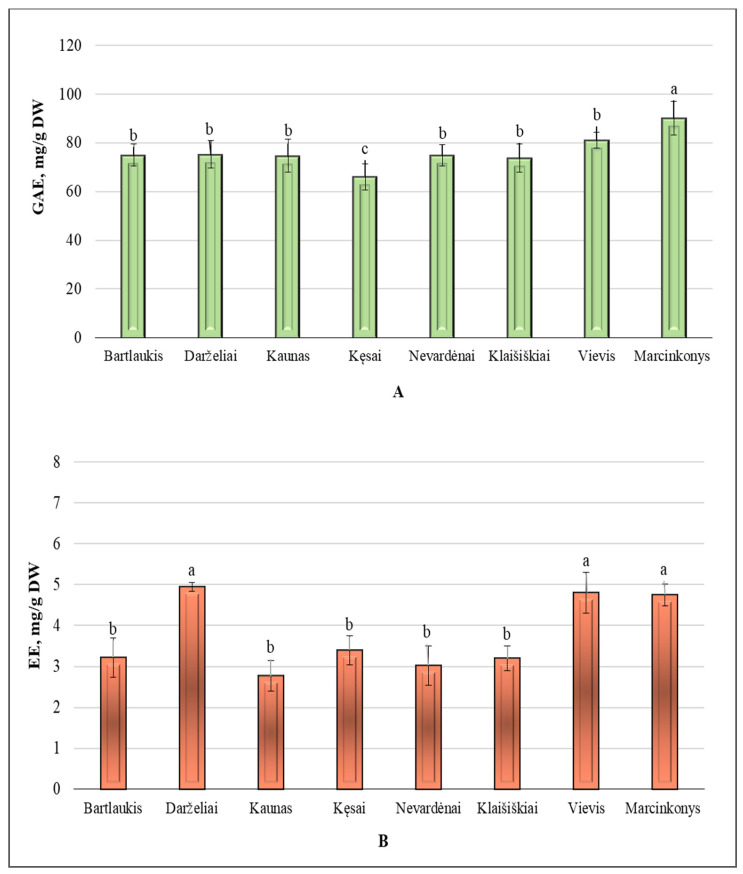
(**A**): variation in the total phenolic compound content (GAE mg/g DW) of *A. spicata* leaf samples from different Lithuanian habitats; (**B**): variation in the total proanthocyanidins content (EE mg/g DW) of *A. spicata* leaf samples from different Lithuanian habitats. Different letters indicate statistically significant differences between habitats (*p* < 0.05).

The total phenolic content in *A. spicata* leaf samples from different Lithuanian habitats is shown in Figure 1A. The average total amount of phenolic compound in the collected samples was 76.37 ± 5.34 GAE mg/g. The results clearly show that the highest content of phenolic compounds (90.17 ± 7.03 GAE mg/g) was found in leaves collected from the Marcinkonys habitat (Varėna district). This content significantly differed from that of the Vievis habitat (81.12 ± 3.13 GAE mg/g), Klaišiškiai habitat (73.79 ± 5.86 GAE mg/g), Nevardėnai habitat (45.01 ± 3.26 GAE mg/g), Kęsai habitat (66.02 ± 5.46 GAE mg/g), Kaunas habitat (74.75 ± 6.75 GAE mg/g), Darželiai habitat (75.24 ± 5.62 GAE mg/g), and Bartlaukis habitat (75.01 ± 4.48 GAE mg/g) (*p* < 0.05). The lowest phenolic compound content (66.02 ± 5.46 GAE mg/g) was found in the Kęsai habitat (Telšiai district) (*p* < 0.05). The coefficient of variation in the total phenolic compounds content was 7.02%. These results indicate that total phenolic levels remain relatively consistent across the habitats studied.

Figure 1B presents the variation in the total amount of proanthocyanidins across different Lithuanian habitats. The average total content of proanthocyanidins in leaf samples collected from various habitats was 3.76 ± 0.35 EE mg/g. The highest content of proanthocyanidins was identified in *A. spicata* leaf samples collected from the forest habitats of Darželiai (4.94 ± 0.10 EE mg/g), Vievis (4.80 ± 0.50 EE mg/g), and Marcinkonys (4.75 ± 0.26 EE mg/g). These results significantly differed from those of leaf samples collected in the Bartlaukis, Kaunas, Kęsai, Nevardėnai, and Klaišiškiai habitats (*p* < 0.05). The content of proanthocyanidins determined in the Kaunas habitat was 1.7 times lower than the highest content found in leaves collected from the Darželiai habitat. The coefficient of variation was 13.5%.

### 2.2. Qualitative and Quantitative Analysis of Phenolic Compounds

Thirty-two individual phenolic compounds belonging to the groups of phenolic acids, flavonols, and flavan-3-ols were identified by HPLC-PDA and HPLC-MS in all tested *A. spicata* leaf samples collected from different habitats (Table 1). The profile of identified phenolic acids comprised neochlorogenic acid, chlorogenic acid, 4-O-caffeoylquinic acid, a caffeoylquinic acid derivative, a hydroxycinnamic acid derivative, coumaric acid, coumaroylquinic acid, 1,5-dicaffeoylquinic acid, and protocatechuic acid. Chlorogenic acid was the predominant compound in leaf samples from different habitats. It constituted approximately 42.00% of the total individual polyphenols in the leaf samples. The coefficient of variation of chlorogenic acid in different habitats was 18.65%. The highest content of chlorogenic acid was found in the leaf samples collected from the Nevardėnai habitat (26,863.77 ± 107.95 µg/g), while the lowest was found in the samples collected from the Marcinkonys habitat (13,337.00 ± 389.11 µg/g). Neochlorogenic acid constituted approximately 13.06% of the total individual polyphenols in the *A. spicata* leaf samples. The coefficient of variation for neochlorogenic acid across different habitats was 38.56%. 4-O-caffeoylquinic and 1,5-dicaffeoylquinic acids made up approximately 2–5% of the total individual polyphenols in the leaf samples. The greatest amounts of 4-O-caffeoylquinic, 1,5-dicaffeoylquinic, and 4-p-coumaroylquinic acids were identified in the *A. spicata* leaf samples collected from the West Lithuanian Outskirts of Bartlaukis and Klaišiškiai, with values of 1086.54 ± 44.23 µg/g, 2816.76 ± 70.01 µg/g, and 311.44 ± 8.93 µg/g, respectively. The contents of coumaric acid and protocatechuic acid were the lowest in the *A. spicata* samples. Coumaric acid and protocatechuic acid made up only about 0.30% of the total individual polyphenols in these samples.

Twenty-two flavonols (rutin, isorhamnetin-3-rutinoside, kaempferol-3-O-rutinoside, isoquercitrin, hyperoside, quercetin-3-arabinoside-7-glucoside, kaempferol-3-sambubioside, quercetin-3-O-robinobioside, isorhamnetin derivative, reynoutrin, kaempferol derivative, astragalin, quercetin-3-O-malonylglucoside, quercetin-3-O-α-L-arabinopyranoside, isorhamnetin-3-O-glucoside, quercitrin, kaempferol-3-O-arabinoside, kaempferol-3-O-acetyl-glucoside, isorhamnetin pentoside, afzelin, quercetin-3-O-acetyl-rhamnoside, kaempferol-3-O-(6-acetyl-galactoside)-7-O-rhamnoside, and one flavan-3-ol ((−)-epicatechin) were identified in the *A. spicata* leaf samples. The predominant flavonol was quercitrin, which accounted for 18.00% of the total phenolic compounds in the leaves of *A. spicata*. On average, the amount of quercitrin in the leaf samples was 20,580.53 µg/g. The amount of quercitrin determined in the leaf samples from the Nevardėnai habitat was 3875.31 ± 65.73 µg/g. These results significantly differed from those of leaf samples collected in the Darželiai, Kaunas, Kęsai, Klaišiškiai, Vievis, and Marcinkonys habitats (*p* < 0.05). The lowest amounts of the identified flavonol group compounds were found of kaempferol derivative and isorhamnetin pentoside, 400.153 µg/g and 436.42 µg/g, respectively.

The highest amounts of the only identified flavan-3-ol, (−)-epicatechin, were found in southern Lithuania: in the habitats of Darželiai (1994.77 ± 60.04 µg/g) and Marcinkonys (1903.47 ± 79.09 µg/g). These results significantly differed from those of leaf samples collected in the Bartlaukis, Kaunas, Kęsai, Klaišiškiai, Vievis, and Nevardėnai habitats (*p* < 0.05). Quercitrin, rutin, hyperoside, quercetin 3-arabinoside 7-glucoside, isoquercitrin, quercetin 3-O-α-L-arabinopyranoside, and (−)-epicatechin, on average, accounted for 78% of the total identified flavonoids.

The total amounts of identified compounds ranged from 28.15 ± 1.38 GAE mg/g (Kaunas) to 59.87 ± 1.17 GAE mg/g (Nevardėnai). The total amounts of phenolic acids and flavonoids showed a strong correlation (R^2^ = 0.92, R = 0.96, *p* < 0.05). Bartlaukis, Nevardėnai, and Klaišiškiai, belonging to western Lithuanian habitats, were determined with the greatest amounts of all identified compounds.

### 2.3. Determination of Radical Scavenging and Reducing Activity of A. spicata Extracts

**Figure 2:**
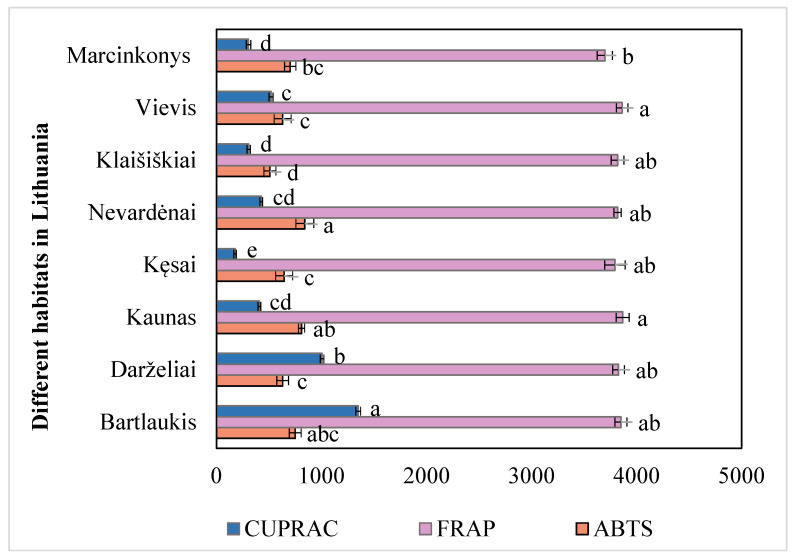
Variation in antioxidant activity (µmol TE/g) of *A. spicata* extracts from different Lithuanian habitats. Different letters indicate statistically significant differences between habitats (*p* < 0.05).

The antioxidant activity of *A. spicata* leaves was evaluated using three assays: FRAP, ABTS, and CUPRAC. The results are presented in Figure 2. The average FRAP assay result for *A. spicata* leaves collected from different habitats was 3817.83 µmol TE/g. Leaf samples from the Kaunas habitat (3868.45 ± 60.78 µmol TE/g) exhibited the highest radical activity and differed significantly from those of the Bartlaukis habitat (3851.99 ± 56.58 µmol TE/g), Darželiai habitat (3828.45 ± 56.58 µmol TE/g), Nevardėnai habitat (3818.98 ± 61.15 µmol TE/g), Kęsai habitat (3794.32 ± 97.91 µmol TE/g), and Marcinkonys habitat (3697.92 ± 72.67 µmol TE/g) (*p* < 0.05). The lowest radical scavenging activity was observed in the leaf samples collected from the Marcinkonys habitat (3697.92 ± 72.67 µmol TE/g). These results differed significantly from those of the Vievis habitat (3862.73 ± 54.26 µmol TE/g) and the Kaunas habitat (3868.45 ± 61.15 µmol TE/g) (*p* < 0.05). The coefficient of variation in the reducing antioxidant activity of *A. spicata* leaf samples collected from different habitats was 1.61%. The results presented in Figure 2 confirm that, upon evaluating the antioxidant activity of *A. spicata* leaf extracts, the statistically significant highest antioxidant activity was determined using the FRAP method.

The average ABTS assay result for *A. spicata* leaves collected from different habitats was 689.43 µmol TE/g. Leaf samples from the Nevardėnai habitat (841.16 ± 85.45 µmol TE/g), Kaunas habitat (809.87 ± 61.15 µmol TE/g), and Bartlaukis habitat (749.47 ± 56.16 µmol TE/g) exhibited the highest radical scavenging activity, significantly differing from those collected in the Darželiai habitat (629.82 ± 56.16 µmol TE/g), Kęsai habitat (644.68 ± 80.75 µmol TE/g), Klaišiškiai habitat (508.36 ± 57.11 µmol TE/g), Vievis habitat (629.79 ± 80.86 µmol TE/g), and Marcinkonys habitat (702.30 ± 54.20 µmol TE/g) (*p* < 0.05). The lowest radical scavenging activity was observed in leaf samples from the Klaišiškiai habitat (508.36 ± 57.11 µmol TE/g). The coefficient of variation in the scavenging activity of *A. spicata* leaf samples collected from different habitats was 9.06%.

The average CUPRAC assay result for *A. spicata* leaves collected from different habitats was 562.33 µmol TE/g. Leaf samples from the Bartlaukis habitat (1349.67 ± 23.05 µmol TE/g) exhibited the highest reducing activity and differed significantly from those of the Darželiai habitat (1005.84 ± 23.05 µmol TE/g), Kaunas habitat (408.53 ± 17.28 µmol TE/g), Kęsai habitat (177.98 ± 13.01 µmol TE/g), Nevardėnai habitat (425.92 ± 12.12 µmol TE/g), Klaišiškiai habitat (306.71 ± 16.31 µmol TE/g), Vievis (519.15 ± 19.06 µmol TE/g), and Marcinkonys habitat (304.86 ± 21.41 µmol TE/g) (*p* < 0.05). The lowest reducing activity was observed in the leaf samples collected from the Kęsai habitat (177.98 ± 13.01 µmol TE/g). The coefficient of variation in the reducing activity of *A. spicata* leaf samples collected from different habitats was 3.00%.

### 2.4. Principal Component Analysis

**Figure 3:**
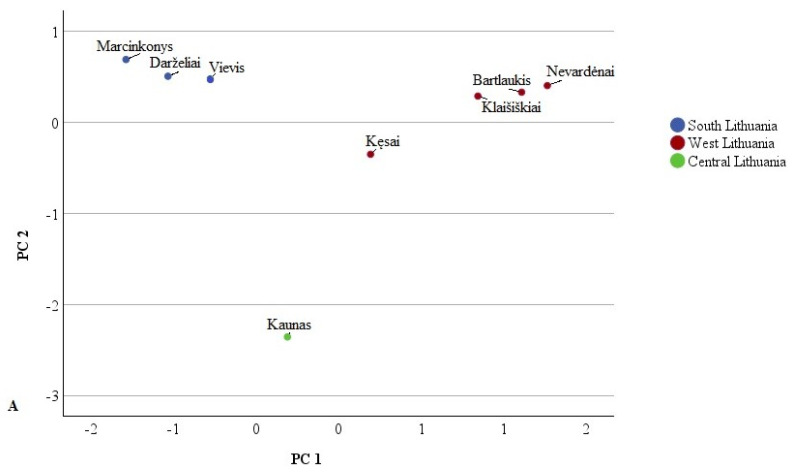

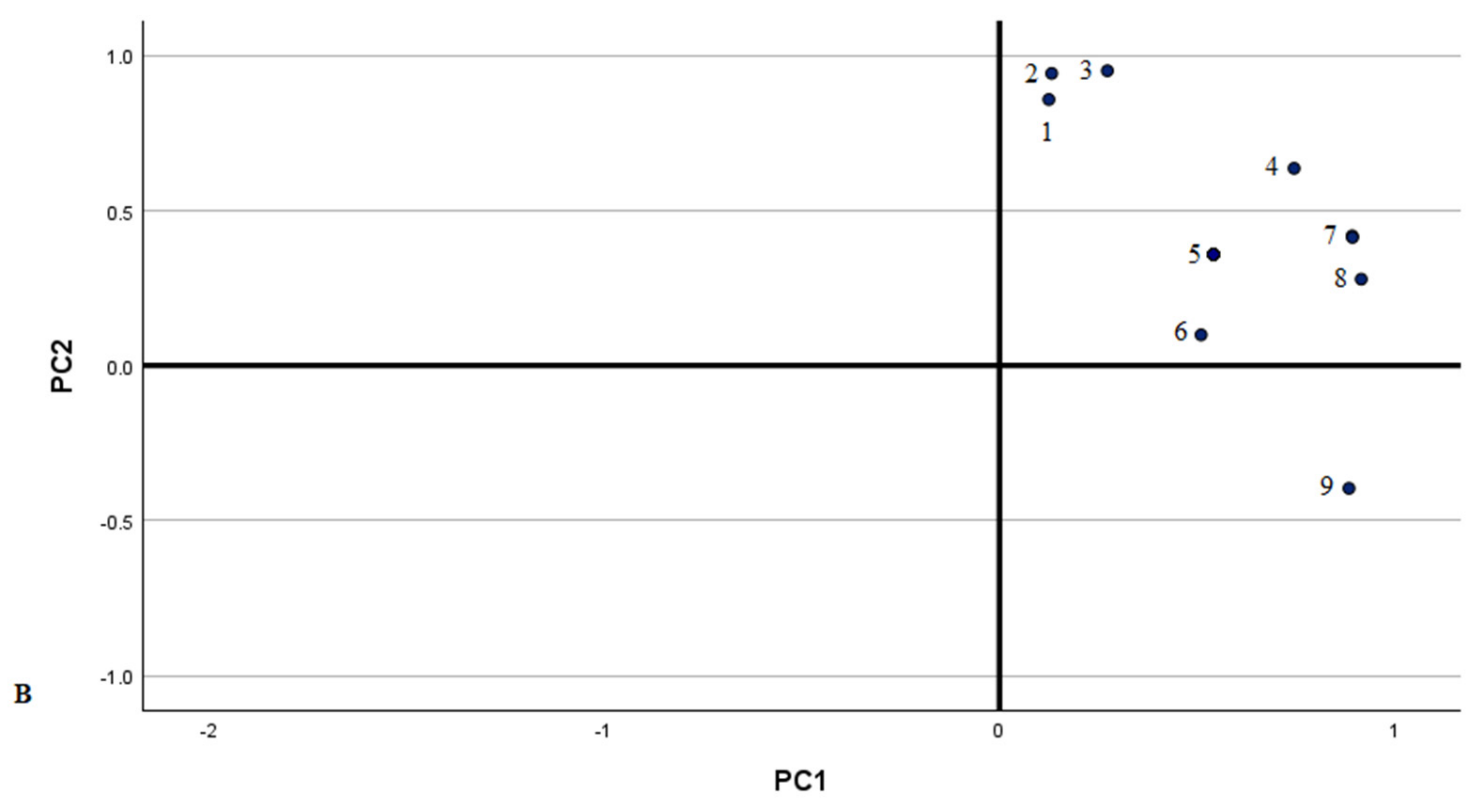
PCA score plots (**A**) and loading plots (**B**) of phenolic acid variables in different Lithuanian regions. 1—Protocatechuic acid; 2—Coumaric acid; 3—Neochlorogenic acid; 4—1,5-dicaffeoylquinic acid; 5—Chlorogenic acid; 6—4-p-coumaroylquinic acid; 7—Hydroxycinnamic acid derivative; 8—Caffeoylquinic acid derivative; 9—4-O-caffeoylquinic acid.

**Figure 4:**
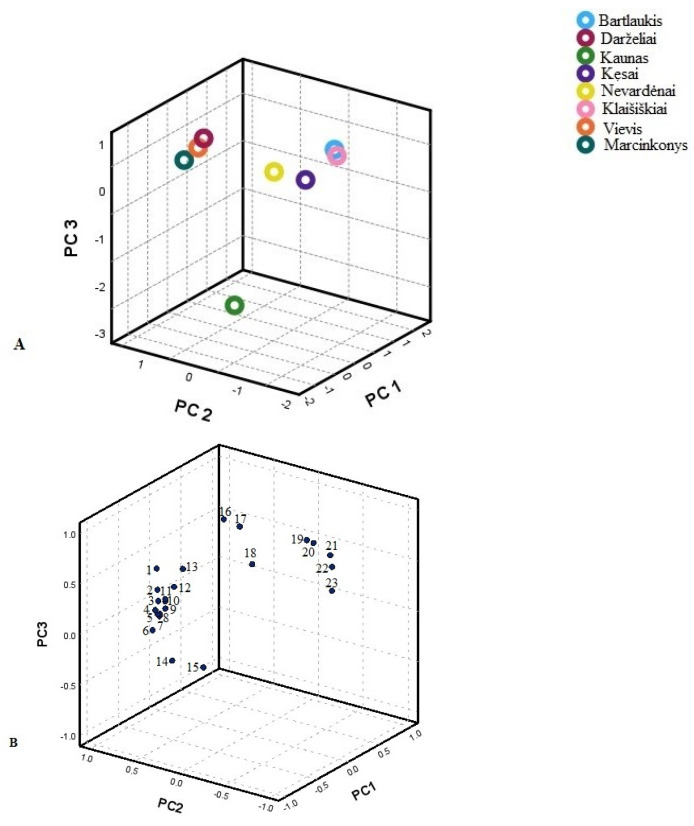
PCA score plots (**A**) and loading plots (**B**) of flavonoid variables in different Lithuanian regions. 1—Quercetin-3-O-robinobioside; 2—Reynoutrin; 3—Isoquercitrin; 4—Hyperoside; 5—Isorhamnetin derivative; 6—Isorhamnetin pentoside; 7—Isorhamnetin-3-rutinoside; 8—Quercetin-3-arabinoside-7-glucoside; 9—Quercetin-3-O-malonylglucoside; 10—Quercetin-3-O-α-L-arabinopyranoside; 11—Rutin; 12—Kaempferol derivative; 13—Quercitrin; 14—Astragalin; 15—Isorhamnetin-3-O-glucoside; 16— Kaempferol-3-O-acetyl-glucoside; 17—Afzelin; 18—Kaempferol-3-O-(6-acetyl-galactoside)-7-O-rhamnoside; 19—(−)-epicatechin; 20—Kaempferol-3-O-arabinoside; 21—Kaempferol-3-sambubioside; 22—Quercetin-3-O-acetyl-rhamnoside; 23—Kaempferol-3-O-rutinoside.

Principal Component Analysis (PCA) was used to isolate the major components and investigate the relationships between phenolic compounds, aiming to identify the primary predictors among the different Lithuanian samples studied. The loadings revealed characteristic variables for the identified phenolic acids and flavonoids, displayed in PCA loading plots illustrating the data along the major components (Figure 3 and Figure 4).

The first PCA model was constructed using identified phenolic acids (Figure 3). Two principal components covering 85.01% of the total data variance were extracted. The first principal component (PC1) explained 45.94% of the total variance. PC1 positively correlated with the amounts of 1,5-dicaffeoylquinic acid (0.979), chlorogenic acid (0.953), 4-p-coumaroylquinic acid (0.951), hydroxycinnamic acid derivative (0.882), and neochlorogenic acid (0.817). The PC2 positively correlated with 4-O-caffeoylquinic acid (0.870) and negatively with coumaric acid (−0.640), protocatechuic acid (−0.580), and neochlorogenic acid (−0.556). Chlorogenic acid predominated in all studied parts of Lithuania.

The second PCA model was constructed using identified flavonoids (Figure 4). Three principal components characterised 95.26% of the total variance. The first principal component explained 60.46% of the total variance and was positively correlated with the amounts of quercetin-3-O-malonylglucoside, isorhamnetin derivative, quercetin-3-O-robinobioside, quercetin-3-O-α-L-arabinopyranoside, hyperoside, quercitrin, isorhamnetin-3-O-glucoside, isorhamnetin pentoside, rutin, reynoutrin, quercetin-3-O-α-L-arabinopyranoside, kaempferol derivative, and isoquercitrin (0.997, 0.994, 0.993, 0.987, 0.984, 0.984, 0.983, 0.964, 0.857, 0.921, 0.897, 0.776, and 0.770, respectively). The second principal component accounted for 17.88% of the total variance and was positively correlated with kaempferol 3-O-(6″-acetyl-galactoside) 7-O-rhamnoside (0.863) and (−)-epicatechin (0.845) and negatively with hyperoside (−0.610). The third principal component was correlated with kaempferol 3-O-acetyl-glucoside (0.737) and astragalin (0.592). The first group coupled habitats in South Lithuania (Marcinkonys, Vievis, and Darželiai). This group was characterised by the greatest amounts of (−)-epicatechin and kaempferol derivatives, namely kaempferol-3-sambubioside, kaempferol-3-rutinoside, kaempferol 3-O-arabinoside, and afzelin, compared to other habitats. Nevertheless, the predominant compound was rutin. The second group contained samples from West Lithuania (Kęsai, Klaišiškiai, Bartlaukis, and Nevardėnai). This group showed the highest amounts of quercetin and isorhamnetin glycosides, with the significant predominant compound being quercitrin. The third group contained samples from Central Lithuania (Kaunas). This group was characterised by the greatest amounts of rutin and quercitrin. The samples from these populations were distributed along the second principal component (PC2) and predominantly on the positive side of the third principal component (PC3). This distribution highlighted a high degree of different habitat variability, indicating significant diversity within the populations regarding the traits or variables analysed.

## 3. Discussion

This study provides a detailed analysis of the phytochemical composition of *Amelanchier × spicata* (Lam.) K. Koch leaf samples collected from natural populations, highlighting significant intrapopulational variability in their chemical profiles. The research demonstrates that the phytochemical composition of *A. spicata* leaves is influenced by a combination of factors, including the specific growing habitat and geographical region. Notably, significant differences in the concentration of various phytochemicals were observed between leaf samples collected from habitats in Lithuania’s western, central, and southern regions. These findings highlight the role of local environmental conditions in shaping the chemical profile of plant species. The *Maloideae* is a subfamily of the plant family *Rosaceae* Juss., within the subtribe *Malinae* Reveal, which includes a variety of fruit-bearing plants, most notably *Malus* Mill., *Pyrus* L., *Cydonia* Mill., *Sorbus* L., *Aronia* J. Mitch., *Chaenomeles* Lindl., *Amelanchier* Medik., and another related genus [26]. Plants of the *Maloideae* C. Weber subfamily are known for their fruits, but the leaves of these plants also contain a considerable variety of biologically active compounds. The plants of the *Maloideae* subfamily, with their diverse phytochemical compositions, have become a focus of increasing interest due to emerging evidence of the strong antioxidant activity of their leaves [27,28,29,30,31]. Liaudanskas et al. determined that the leaves of different *Malus* cultivars are a source of phenolic compounds [27,28]. Studies have shown that apple leaf extracts’ total amount of phenolic compounds ranges from 98.81 mg GAE/g DW to 163.35 mg GAE/g DW [27]. Thi and Hwang compared the total variation of phenolic compounds in *Aronia melanocarpa* (Michx.) Elliott leaves and found that the total phenolic content in 80% ethanol leaf extracts collected at different stages ranged from 139.3 mg GAE/g to 141.6 mg GAE/g [32]. The study by Owczarek et al. confirms that individual phenolic compounds found in *A. melanocarpa* leaves significantly impact antioxidant and cytotoxic activity [33]. Olszewska et al. found that the leaves and inflorescences of *Sorbus* L. contained higher amounts of total phenolic content than the fruits. The highest total amount of phenolic compounds was reported in the dried leaves of *S. wilfordii* (12.31% GAE) and in the inflorescences of *S. aucuparia* (11.83% GAE) [34]. In our study, the total content of phenolic compounds in ethanolic *A. spicata* leaf extracts ranged from 66.02 mg GAE/g DW to 90.17 mg GAE/g DW across different habitats. These findings indicate that while *A. spicata* leaves have slightly lower phenolic content compared to certain well-studied species, their levels are comparable to those of other underutilised species within the *Maloideae*. *A. spicata* leaves are notable for their predominance of chlorogenic and neochlorogenic acids, which are key contributors to antioxidant activity. These compounds, which are also abundant in *Malus* spp., have well-documented health benefits, including anti-inflammatory and cardioprotective effects [35,36]. While species such as *Malus* are widely cultivated and utilised, *A. spicata* represents a valuable, underutilised resource that can provide similar bioactive compounds, particularly in regions where it grows invasively [3,16]. Therefore, data suggest positioning this species as a potential alternative for nutraceutical or pharmaceutical products focused on antioxidant and anti-inflammatory benefits. Some differences were observed when comparing the results of this study with data published by other researchers. As reported by Męczarska et al., the leaves of *A. alnifolia* demonstrated a total polyphenol content of 185.23 mg GAE/g DW, which was 2.5 times higher compared to our result [23]. These discrepancies may be attributed to variations in the species within the *Maloideae* subfamily, as different species can exhibit distinct biochemical compositions, environmental adaptations, and metabolic pathways that influence the concentration and composition of phenolic compounds. Additionally, factors such as geographical location, climate, raw material drying temperature, and the extraction method may further contribute to these differences [37].

The western and southern habitats showed the greatest differences in the profiles of phenolic compounds. Southern habitats were characterised by higher average monthly temperatures and varying precipitation levels from lower in April and July to higher in the August-September seasons. Furthermore, different soil types and higher soil pH were characteristic of western habitats, contributing to greater amounts of phenolic acids and individual flavonoids. Hosamani et al. determined that soil pH significantly impacts the accumulation of secondary metabolites and impacts plant growth, as the higher pH levels decrease the bioavailability of micronutrients [38]. Secondary metabolites, particularly phenolic compounds, serve as plant defence mechanisms, coping with biotic and abiotic stress and redox reactions in soils. Furthermore, these compounds act as allelochemicals [39]. Callaway et al. determined that flavonoids were the key contributors to the invasive potential of *Alliaria petiolata* (M.Bieb.) Cavara and Grande in North America, acting by inhibiting mycorrhizal functions in native plants [40]. The spread of invasive trees in Europe poses significant ecological, economic, and social challenges. Proactive management, ecological restoration, international cooperation, and community involvement and awareness are essential to mitigate their impact and protect Europe’s native species [41]. Scientific studies have shown that the leaves of invasive trees, such as *Quercus rubra* L., *Ailanthus altissima* (Mill.) Swingle, *Acer negundo* L., and *Robinia pseudoacacia* L., can significantly impact ecosystems. These leaves alter soil composition by releasing chemicals that inhibit the growth of native plants and promote an increase in nitrogen concentration. This not only reduces the diversity of native plant species but also disrupts the natural nutrient cycles in the soil [42,43,44,45,46]. The study revealed significant phytochemical variability across different habitats, influenced by environmental factors such as soil type, pH, and light exposure. Samples from forest habitats, such as Marcinkonys and Vievis, were characterised by higher amounts of kaempferol derivatives and epicatechin content, likely due to forest-specific conditions such as partial shading and reduced abiotic stress. In contrast, samples from outskirt habitats, such as Kęsai and Bartlaukis, exhibited higher concentrations of hydroxycinnamic acids, reflecting adaptations to higher light intensity and potentially nutrient-depleted soils. These findings suggest that habitat-specific factors play a critical role in determining the phytochemical profile of *A. spicata*, and targeted harvesting strategies might optimise extract composition.

Chlorogenic acid was identified as the predominant bioactive compound in the leaves of *A. spicata*. As various studies have shown, chlorogenic acid neutralises free radicals in the body, protecting cells from oxidative stress and reducing the risk of chronic diseases such as heart disease, cancer, and neurodegenerative conditions like Alzheimer’s [47]. This phenolic acid is well known for its antioxidant, anti-inflammatory, and potential health-promoting properties, making it a significant constituent in the *Maloideae* subfamily plants phytochemical profile [33,34,36,37,48,49,50,51,52,53]. The high concentration of chlorogenic acid in *A. spicata* leaves suggests that the plant could be a valuable natural source for nutraceutical and medicinal applications, particularly for conditions where oxidative stress and inflammation are involved. Additionally, its prevalence might influence the pharmacological activity of *A. spicata* and contribute to its traditional therapeutic uses. Both chlorogenic and neochlorogenic acids are the predominant compounds in plant species of the subtribe *Malinae* [54,55,56]. The phenolic profile of *A. spicata* leaves differs markedly from that of the fruits reported in the literature [23]. The fruits are rich in anthocyanins, including cyanidin-3-galactoside and cyanidin-3-glucoside, alongside notable levels of sugars and organic acids, which contribute to their distinct nutritional and sensory qualities [57]. In contrast, the leaves exhibit a higher abundance of flavonoids, particularly quercetin derivatives, such as quercetin-3-galactoside, quercetin-3-robinobioside, and quercetin-3-vicianoside [23]. These compounds are well recognised for their antioxidant, anti-inflammatory, and potential health-promoting properties [58,59]. *A. spicata* leaves could serve as a complementary or alternative source of bioactive compounds, particularly in formulations targeting oxidative stress-related conditions. While fruits may remain the preferred source for direct consumption and flavour-related applications, leaves hold promise for inclusion in nutraceuticals and dietary supplements where flavonoid-derived compounds are the primary focus [24,60,61].

Lavola et al. determined that *A. alnifolia* leaves consisted of quercetin 3-galactoside and 3-glucoside, (−)-epicatechin, and chlorogenic acid, which were the main phenolics in the leaves of all cultivars [18]. Męczarska et al. found that *A. alnifolia* fruits accumulate 10.0 times more chlorogenic acid than the leaves. Lower amounts of other hydroxycinnamic acids were determined, such as 4-O-caffeoylquinic acid, 1,5-dicaffeoylquinic acid, neochlorogenic acid, 4-p-coumaroylquinic acid, and coumaric acid [23]. Compared to *A. alnifolia*, a closely related species, *A. spicata* leaves exhibit a higher proportion of hydroxycinnamic acids relative to flavonols. This differentiation may reflect adaptations to local environmental conditions and underscores the importance of studying regional populations of invasive species. Our identified compounds from the flavonol group – quercitrin, rutin, hyperoside, quercetin 3-arabinoside 7-glucoside, isoquercitrin, and quercetin 3-O-α-L-arabinopyranoside – exhibit diverse biological activities beneficial for human health. Rutin and quercitrin exhibit antiviral activity against various viruses and inhibit the growth of harmful bacteria, supporting a robust immune system [62]. Rutin also promotes venous health and alleviates varicose veins [63]. Hyperoside protects against oxidative stress-related damage in the brain, heart, and pancreas, while isoquercitrin may help regulate blood sugar and support cognitive health [64,65]. As different studies have shown, quercetin glucosides are potent bioactive compounds that can benefit human health in numerous ways, from reducing inflammation to improving heart and brain health [66]. Quercetin 3-arabinopyranoside is the major compound isolated from *Alchemilla xanthochlora* Rothm., used in European traditional medicine as an astringent against bleeding and diarrhoea [67]. Cho et al. determined that *M. domestica* fruits accumulate quercetin 3-arabinopyranoside, which could be a natural soluble epoxide hydrolase inhibitor both in vitro and in silico [68]. It has been reported that flavan-3-ols, specifically (+)-catechin and (−)-epicatechin, are the primary phenolic compounds in *Malus* species, accounting for 55–85% of the total phenolic content [69]. In our study, the total amount of (−)-epicatechin across different habitats ranged from 1045.85 µg/g to 1994.77 µg/g. The study by Li et al. confirmed the influence of genetic background, growth latitude, and bagging treatment on (−)-epicatechin in the fruits of cultivars and wild types of *Malus* sp. [70]. Studies on the biological activity of epicatechin highlight its potential to enhance physical performance, aid muscle recovery, and mitigate exercise-induced muscle damage, thanks to its antioxidant and anti-inflammatory properties. Epicatechin has also been linked to improved cardiovascular health by boosting blood flow, lowering blood pressure, and supporting healthy endothelial function [71]. Additionally, it is recognised for its role in enhancing muscle performance and recovery, making it a favoured supplement among athletes. Emerging research further suggests that epicatechin may possess neuroprotective benefits, potentially improving cognitive health and reducing the risk of neurodegenerative diseases [72]. The presence of this compound in *A. spicata* leaves positions the species as a potential contributor to formulations targeting these health outcomes, complementing the use of *Malus* and other traditional sources.

Antioxidant activity in plants of the *Maloideae* subfamily has garnered considerable interest due to the high levels of biologically active compounds, particularly phenolic compounds, that these plants contain. Commonly found in fruits, leaves, and other tissues, compounds such as chlorogenic acid, quercetin, and proanthocyanidins contribute to the strong antioxidant properties observed across various *Maloideae* species. The bioactive profile of *Maloideae* plants, along with their high antioxidant potential, positions them as valuable sources of natural ingredients for nutraceutical and pharmaceutical applications. Raudone et al., using the post-column FRAP assay, determined that the leaves of different *Sorbus* L. species (*S. commixta*, *S. discolor*, and *S. gracilis*) exhibited significantly higher total antioxidant activity, with values of 175.30 μmol TE/g DW, 169.20 μmol TE/g DW, and 148.11 μmol TE/g DW, respectively [73].

Various studies have confirmed the outstanding antioxidant activity of *Malus* L. leaves due to their rich and diverse phenolic content [36,74]. Sowa et al. determined that apple extracts could be prospective antioxidant and antimicrobial agents [75]. In our study, *A. spicata* leaves exhibited significant antioxidant activity, as evidenced by the results of the ABTS, FRAP, and CUPRAC assays, correlating strongly with the identified phenolic compounds. The results support that *A. spicata* is not only an alternative to traditional sources but also a valuable addition to sustainable and functional applications. Furthermore, specific plants within the *Maloideae* subfamily, including *Amelanchier* species, could serve as promising sources of biologically active compounds with potent antioxidant activity. Didur et al. determined that the fresh fruits of *A. humilis* contain more phytochemical components (total polyphenol content, total flavonoid content) with stronger antioxidant properties than *A. alnifolia* and *A. canadensis* [76]. Carotenoids found in *Amelanchier alnifolia* fruits exhibit significant antioxidant activity. They help neutralise free radicals, thereby reducing oxidative stress and protecting cells from damage. This antioxidant property contributes to the overall health benefits of these fruits, supporting cellular integrity and potentially reducing the risk of chronic diseases associated with oxidative damage [20].

The PCA plotting revealed particular distribution patterns of *A. spicata* populations due to possible differences in the location and type of habitats. The distinguished groups were with the specific chemical compositions of interest. Our results revealed significant variations and elucidated three regions with notable amounts of predominant compounds: chlorogenic acid, (−)-epicatechin, quercitrin, rutin, and specific consistent patterns of minor flavonoids, particularly kaempferol and isorhamnetin derivatives. The three forest habitats, namely Vievis, Marcinkonys, and Darželiai, were distinguished from the remaining outskirt habitats. Plants growing in forest environments might exhibit different phenolic profiles than those growing on the outskirts due to variations in light intensity, soil nutrients, and competition [77]. Forest plants typically grow in part shade, which can reduce the total amounts of flavonoids and phenolic acids [78]. Shade tolerance might be one of the feature characteristics of invasive plants and promote their superiority in the habitat [79]. On the other hand, plants growing on the outskirts, which experience higher light exposure, might turn the production of phytochemicals towards antioxidant-active compounds [80]. Our research confirmed that leaf samples from forest habitats contained a more diverse quantitative profile of flavonoids compared to samples collected in the outskirts. On the other hand, samples from outskirt habitats tend to have higher concentrations of hydroxycinnamic acids, reflecting adaptations to the environmental conditions. Furthermore, outskirt habitats often have soil compositions influenced by human activities, which can modify the phytochemical content through changes in nutrient availability and microbial interactions [81,82].

## 4. Materials and Methods

### 4.1. Plant Material

**Figure 5:**
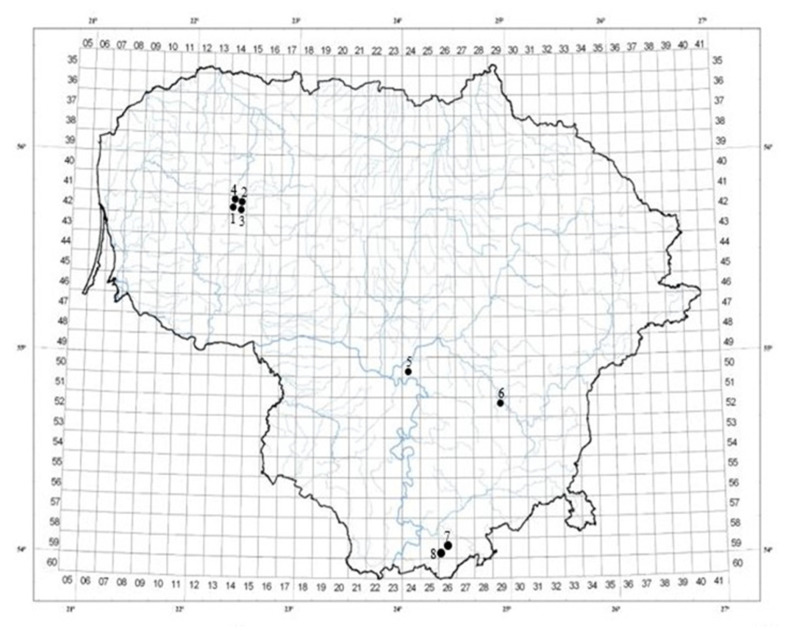
Locations of *A. spicata* leaf sampling sites in Lithuania.

*A. spicata* leaves were collected from eight different habitats in Lithuania, with ten randomly selected bushes from each habitat, in July 2023 (Figure 5, Table 2). The plant species was identified based on morphological characteristics by Sandra Saunoriūtė. Most forests were dominated by Scots pine (*Pinus sylvestris* L.); however, Nevardėnai and Kęsai were characterised by Silver birch (*Betula pendula* Roth) as the main tree species. The leaves were dried at +25 °C in a well-ventilated chamber, protected from direct sunlight and moisture. The loss on drying was evaluated according to the European Pharmacopoeia [83].

The meteorological data, including the average air temperature (°C), precipitation (mm), and sunshine duration (h), along with soil characteristics from the study sites, were sourced from the Lithuanian Hydrometeorological Service and the Soil Atlas of Europe [84,85]. These data are detailed in Tables S1–S3. Lithuania’s climate falls under the Dfb classification, characterised by a continental climate with snowy winters, fully humid conditions, and warm summers. Summer temperatures typically range from 21–32 °C during the day and 10–18 °C at night, while winter temperatures range from −12 to 7 °C during the day and −23 to −4 °C at night [86].

### 4.2. Chemicals and Solvents

Ethanol (96%) was obtained from AB Vilniaus Degtinė (Vilnius, Lithuania). Folin–Ciocalteu reagent, acetic acid, hydrochloric acid, sodium carbonate, gallic acid monohydrate, ABTS (2,2′-azino-bis(3-ethylbenzothiazoline-6-sulfonic acid)), Trolox ((+)-6-hydroxy-2,5,7,8-tetramethylchroman-2-carboxylic acid), copper(II) chloride dihydrate, potassium persulfate, sodium acetate, TPTZ (2,4,6-tripyridyl-s-triazine), DMAC (4-dimethylaminocinnamaldehyde), neocuproine, hydrochloric acid, ammonium acetate, ferric(III) chloride hexahydrate, neochlorogenic acid, chlorogenic acid, 4-caffeoylquinic acid, coumaric acid, 5-caffeoylshikimic acid, 1,5-dicaffeoylquinic acid, protocatechuic acid, quercetin, rutin, kaempferol, hyperoside, isoquercitrin, isorhamnetin-3-rutinoside, reynoutrin, astragalin, quercitrin, afzelin, (−)-epicatechin, procyanidin B2, and procyanidin C1 were purchased from Sigma–Aldrich (Buchs, Switzerland). Distilled water was purified using a Milli-Q system (Millipore, Bedford, MA, USA).

### 4.3. Preparation of Extracts

*A. spicata* leaf extracts were prepared using 0.20 g of dried, crushed leaves with 20 mL of 70% ethanol. The samples were extracted in an Elmasonic P ultrasonic bath (Singen, Germany) for 15 min. Subsequently, the extracts were centrifuged in a Biofuge centrifuge (Hanau, Germany) at 8500 rpm for 15 min and then filtered through 0.45 μm membrane filters (Carl Roth GmbH, Karlsruhe, Germany). The samples were stored in a dark, light-protected place. Before injection into the high-performance liquid chromatography (HPLC) system, the extracts were filtered through 0.22 µm pore-size membrane filters and transferred to dark glass vials. Extraction procedures were conducted in triplicate (n = 3) for each sample.

### 4.4. Evaluation of Bioactive Compounds

The total phenolic content (TPC) was determined using the Folin–Ciocalteu assay proposed by Slinkard and Singleton [87], with minor modifications described by Kaunaite et al. [88]. The total phenolic content was calculated from a gallic acid calibration curve (y = 11.157x + 0.0637; R^2^ = 0.9901) and expressed as mg GAE/g DW. The total content of proanthocyanidins (DMAC—4-Dimethylaminocinnamalaldehyde) was determined using the described methodology by Prior et al. [89], calculated from an epicatechin calibration curve (y = 5.5865x − 0.0423; R^2^ = 0.9957) and expressed as mg EE/g DW.

### 4.5. Evaluation of Antioxidant Activity

The ABTS radical cation decolorisation assay was applied according to the methodology described by Re et al. [90], with minor modifications as described by Raudone et al. [91]. A volume of 3 mL of the solution (with an absorbance of 0.800 ± 0.02) was mixed with 20 μL of the tested extract. The decrease in absorbance was measured at a wavelength of 734 nm after the samples were kept in a dark place for 1 h.

The Cupric Ion Reducing Antioxidant Capacity (CUPRAC) assay was performed using the methodology reported by Apak et al. [92]. The working CUPRAC solution included copper (II) chloride (0.01 M in water), ammonium acetate buffer (0.001 M, pH 7), and neocuproine (0.0075 M in ethanol) in a 1:1:1 ratio. During the evaluation, 3 mL of the CUPRAC reagent was mixed with 20 µL of the extract. An increase in absorbance was recorded after 1 h at a wavelength of 450 nm.

The Ferric-Reducing Antioxidant Power (FRAP) assay, established by Benzie and Strain [93], was carried out on the leaf extracts, as reported by Raudone et al. [94], with minor modifications. The working FRAP solution included 2,4,6-tri-2-pyridinyl-1,3,5-triazine (TPTZ) (0.01 M dissolved in 0.04 M HCl), FeCl₃·6H₂O (0.02 M in water), and acetate buffer (0.3 M, pH 3.6) in a ratio of 10:1:1. A volume of 3 mL of the freshly prepared FRAP reagent was mixed with 20 μL of the tested extract. An increase in absorbance was recorded after 1 h at a wavelength of 593 nm.

The antioxidant capacity of the tested extracts was calculated from the Trolox calibration curve and expressed as μmol Trolox equivalent (TE) per gram. TE was calculated according to the following formula:

TE = (C × V)/m (μmol/g)

C: TE concentration of Trolox established from the calibration curve (in μM); V: the volume of the extract (in L); m: the weight of herbal material (in g).

### 4.6. Qualitative and Quantitative Analysis of Phenolic Compounds in A. spicata Leaf Samples

The HPLC-PDA analysis of dwarf serviceberry leaf extracts was performed using a “Waters 2695 Alliance system” (Waters, Milford, MA, USA) with a photodiode array detector “Waters 2998”. According to the HPLC-PDA method by Raudone et al., 2017 [95]. Separation was performed using an ACE (ACT, Aberdeen, UK) (column (C18, 150 mm × 4.6 mm, particle size 3 μm). The mobile phase of the optimised chromatographic method consisted of eluent A (0.05% trifluoroacetic acid) and B (acetonitrile). The gradient was: 0–5 min—12% B, 5–50 min—12–30% B, 50–51 min—30–90% B, 51–56 min—90% B, 57 min—12% B. Eluent flow rate—0.5 mL/min, injection volume—10 μL. The column was temperature–controlled and maintained at +15 °C.

Chromatographic peak identification was performed based on the retention times of analytes and reference compounds, as well as by comparing their UV absorption spectra using a diode array detector. Phenolic acid content was measured at a wavelength of 320 nm, flavonol content at 360 nm, and dihydrochalcone and procyanidin content at 280 nm. The results were adjusted to reflect values for absolutely dry raw material (DW).

The HPLC-MS system comprised a Shimadzu Nexera X2 LC-30AD HPLC system (Shimadzu, Tokyo, Japan) equipped with an LCMS-2020 mass spectrometer (Shimadzu, Tokyo, Japan). Chromatographic separation was performed on the column, and HPLC-PDA analysis conditions were as indicated above. The optimum ESI conditions were set as 350 °C for the interface temperature, 250 °C for the DL temperature, 400 °C for the heat block temperature, 1.5 L/min for the nebulising gas flow, and 10 L/min for the drying gas flow. Positive and negative ion measurements were performed while alternating between positive and negative ionisation modes. The *m*/*z* ranges for positive and negative modes were 50–2000, with a scan speed positive at 5000 u/s, negative ionisation at 15,000 u/s, and 0.1 *m*/*z* steps. Compounds in the sample were identified by comparing the mass spectra obtained with the literature data and mechanisms presented in freely available databases. [96,97,98,99,100,101,102,103,104,105].

### 4.7. Statistical Analysis

Statistical analysis was performed using Microsoft Office Excel 2016 (Microsoft, Redmond, WA, USA) and SPSS Statistics 27 (IBM, Armonk, NY, USA). The experiments were performed in triplicate. The study results were expressed as mean ± standard deviation (SD). Correlations were tested by using the Spearman correlation test. One-way analysis of variance (ANOVA) using the Tukey post hoc criterion was used to assess the statistical significance of the data obtained. Principal component analysis (PCA) was used. PCA factors with eigenvalues greater than 1 were used. The difference was considered statistically significant at *p* < 0.05.

## 5. Conclusions

This study presents the results of investigations into the content of biologically active compounds in the leaves of invasive *Amelanchier × spicata* (Lam.) K. Koch, along with their antioxidant activity. The determined phytochemical and antioxidant activity values show significant variability depending on the geographical region and habitat characteristics. Chlorogenic acid and neochlorogenic acids, as well as the triplet or quercitrin, rutin, and hyperoside, were determined as predominant compounds in the phenolic content. The antioxidant activity of extracts supports the potential of *A. spicata* leaves from outskirt habitats as a natural source of antioxidants for the development of functional ingredients for dietary supplements, pharmaceuticals, and functional foods. The scientific uncertainties to be solved should be directed to the research on the effects of seasonal and environmental factors on phytochemical composition and phenolic fraction biological activity evaluation targeted towards sustainable development and biomedical innovation.

## Figures and Tables

**Table 1:** Variation of qualitative and quantitative amounts of phenolic compounds in different *A. spicata* habitats µg/g DW).

Compound	Bartlaukis Habitat	Darželiai Habitat	Kaunas Habitat	Kęsai Habitat	Nevardėnai Habitat	Klaišiškiai Habitat	Vievis Habitat	Marcinkonys Habitat
Hydroxycinnamic acids								
Neochlorogenic acid	6523.45 ± 320.88 ^a^	6000.86 ± 395.19 ^b^	3295.36 ± 189.15 ^e^	5617.97 ± 338.49 ^d^	6560.47 ± 350.23 ^a^	6091.35 ± 145.77 ^a^	6290.30 ± 305.72 ^a^	5913.21 ± 159.72 ^c^
Chlorogenic acid	26,605.10 ± 508.67 ^a^	14,522.60 ± 435.57 ^d^	10,972.57 ± 424.74 ^f^	16,266.00 ± 418.04 ^c^	26,863.77 ± 107.95 ^a^	25,200.60 ± 500.60 ^b^	14,668.30 ± 283.42 ^d^	13,337.00 ± 389.11 ^e^
4-O-caffeoylquinic acid	1086.54 ± 44.23 ^a^	956.57 ± 52.77 ^b^	1060.59 ± 40.34 ^a^	1066.94 ± 47.07 ^a^	1076.15 ± 75.39 ^a^	1048.43 ± 34.77 ^a^	998.05 ± 40.74 ^b^	938.50 ± 15.42 ^b^
Caffeoylquinic acid derivative	195.18 ± 32.68 ^b^	177.98 ± 4.88 ^b^	183.91 ± 19.14 ^b^	168.43 ± 20.17 ^b^	258.99 ± 45.72 ^a^	162.58 ± 9.04 ^b^	176.36 ± 23.56 ^b^	175.69 ± 10.91 ^b^
Hydroxycinnamic acid derivative	161.37 ± 10.33 ^a^	59.80 ± 1.49 ^c^	48.30 ± 1.64 ^d^	135.25 ± 8.57 ^b^	147.40 ± 13.87 ^b^	164.41 ± 5.52 ^a^	68.06 ± 2.15 ^c^	54.25 ± 2.32 ^d^
Coumaric acid	104.53 ± 4.73 ^b^	105.84 ± 4.27 ^b^	14.19 ± 1.89 ^d^	50.27 ± 2.10 ^c^	113.93 ± 3.06 ^a^	101.89 ± 5.32 ^b^	99.14 ± 3.77 ^b^	102.59 ± 2.49 ^b^
4-p-coumaroylquinic acid	301.26 ± 14.75 ^b^	157.20 ± 6.64 ^e^	110.64 ± 3.68 ^e^	221.50 ± 9.09 ^c^	293.81 ± 5.97 ^b^	311.44 ± 8.93 ^a^	166.28 ± 5.20 ^d^	144.29 ± 4.41 ^e^
1,5-dicaffeoylquinic acid	2715.44 ± 61.60 ^a^	2135.88 ± 54.84 ^b^	1595.64 ± 61.02 ^c^	2130.53 ± 42.91 ^b^	2782.67 ± 64.06 ^a^	2816.76 ± 70.01 ^a^	2145.49 ± 20.13 ^b^	2101.20 ± 70.71 ^b^
Hydroxybenzoic acids								
Protocatechuic acid	60.96 ± 4.84 ^a^	53.55 ± 3.76 ^b^	19.81 ± 1.23 ^c^	67.55 ± 6.27 ^a^	60.05 ± 5.85 ^a^	53.70 ± 4.46 ^b^	60.82 ± 4.44 ^a^	62.14 ± 5.50 ^a^
Flavonols								
Quercetin-3-arabinoside-7-glucoside	2137.10 ± 37.35 ^a^	1587.66 ± 12.44 ^c^	974.35 ± 7.66 ^e^	1245.34 ± 38.13 ^d^	2138.92 ± 34.20 ^a^	1955.98 ± 40.82 ^b^	1607.55 ± 53.72 ^c^	1582.67 ± 45.16 ^c^
Rutin	3264.67 ± 111.52 ^a^	2393.87 ± 30.67 ^b^	1630.48 ± 26.10 ^e^	2014.41 ± 18.02 ^d^	3258.35 ± 154.69 ^a^	3133.20 ± 109.09 ^a^	2437.98 ± 26.98 ^b^	2277.93 ± 40.27 ^c^
Kaempferol-3-sambubioside	260.28 ± 17.82 ^d^	476.14 ± 19.89 ^b^	358.05 ± 7.48 ^c^	347.93 ± 22.32 ^c^	280.44 ± 12.31 ^d^	231.06 ± 11.53 ^e^	506.48 ± 4.35 ^a^	512.13 ± 6.56 ^a^
Quercetin-3-O-robinobioside	364.33 ± 26.51 ^a^	204.11 ± 4.69 ^c^	140.16 ± 1.75 ^d^	193.38 ± 13.79 ^c^	369.52 ± 11.89 ^a^	338.65 ± 13.71 ^b^	205.37 ± 7.33 ^c^	197.36 ± 6.49 ^c^
Hyperoside	3198.22 ± 146.80 ^a^	1308.28 ± 9.12 ^b^	1160.68 ± 30.26 ^c^	1253.61 ± 43.37 ^c^	3140.86 ± 25.77 ^a^	3099.96 ± 87.26 ^a^	1371.32 ± 49.62 ^b^	1332.23 ± 22.31 ^b^
Isoquercitrin	2040.15 ± 38.62 ^b^	805.99 ± 8.72 ^e^	1051.16 ± 5.18 ^c^	813.24 ± 2.77 ^e^	2211.98 ± 30.59 ^a^	2062.15 ± 15.70 ^b^	885.62 ± 30.91 ^d^	822.64 ± 11.97 ^e^
Isorhamnetin-3-rutinoside	634.05 ± 8.15 ^a^	149.07 ± 3.82 ^c^	104.03 ± 9.73 ^e^	143.35 ± 16.56 ^d^	562.59 ± 27.26 ^b^	553.60 ± 16.97 ^b^	174.26 ± 12.40 ^c^	165.58 ± 5.00 ^c^
Kaempferol-3-O-rutinoside	205.17 ± 9.36 ^e^	539.02 ± 19.69 ^a^	470.99 ± 13.39 ^b^	418.28 ± 2.87 ^c^	279.05 ± 14.04 ^d^	173.13 ± 33.81 ^e^	526.26 ± 25.76 ^a^	560.36 ± 33.04 ^a^
Isorhamnetin derivative	238.93 ± 15.90 ^b^	104.30 ± 7.89 ^d^	88.54 ± 7.24 ^e^	103.06 ± 6.30 ^d^	255.70 ± 12.17 ^a^	217.59 ± 9.09 ^c^	106.42 ± 3.06 ^d^	100.09 ± 4.52 ^d^
Reynoutrin	127.82 ± 5.57 ^b^	80.70 ± 2.81 ^c^	60.67 ± 1.52 ^e^	72.28 ± 3.47 ^d^	192.61 ± 5.38 ^a^	126.35 ± 5.01 ^b^	80.87 ± 1.96 ^c^	74.87 ± 2.49 ^c^
Kaempferol derivative	57.59 ± 4.80 ^b^	40.09 ± 2.94 ^d^	39.32 ± 3.09 ^d^	24.43 ± 3.06 ^e^	89.81 ± 7.48 ^a^	55.03 ± 3.09 ^b^	47.90 ± 1.08 ^c^	45.95 ± 1.79 ^c^
Astragalin	106.24 ± 2.79 ^c^	77.02 ± 2.12 ^e^	162.45 ± 3.12 ^a^	78.73 ± 8.75 ^e^	134.64 ± 10.63 ^b^	93.92 ± 7.89 ^d^	70.45 ± 3.56 ^e^	80.58 ± 6.16 ^e^
Quercetin-3-O-malonylglucoside	407.03 ± 11.26 ^a^	156.63 ± 15.19 ^d^	125.94 ± 6.88 ^e^	186.75 ± 10.12 ^c^	421.91 ± 22.02 ^a^	382.22 ± 12.53 ^b^	165.54 ± 4.35 ^c^	157.60 ± 6.71 ^d^
Quercetin-3-O-α-L-arabinopyranoside	1676.84 ± 4.90 ^a^	906.52 ± 14.41 ^b^	658.60 ± 36.83 ^c^	858.09 ± 36.27 ^b^	1642.56 ± 139.98 ^a^	1668.88 ± 58.93 ^a^	907.66 ± 34.41 ^b^	868.40 ± 25.58 ^b^
Isorhamnetin-3-O-glucoside	48.66 ± 4.90 ^c^	36.48 ± 3.00 ^d^	114.67 ± 14.20 ^a^	31.91 ± 5.37 ^d^	57.08 ± 9.32 ^b^	57.08 ± 9.32 ^b^	44.89 ± 6.61 ^c^	44.92 ± 4.13 ^c^
Quercitrin	3785.15 ± 112.13 ^a^	2106.53 ± 53.62 ^c^	1323.20 ± 63.15 ^f^	1745.62 ± 31.16 ^e^	3875.31 ± 65.73 ^a^	3730.92 ± 108.29 ^b^	2080.22 ± 69.33 ^c^	1933.55 ± 55.32 ^d^
Kaempferol-3-O-arabinoside	144.77 ± 9.79 ^b^	229.87 ± 14.55 ^a^	154.65 ± 8.37 ^b^	139.70 ± 10.06 ^b^	153.28 ± 15.81 ^b^	123.16 ± 18.91 ^c^	249.93 ± 8.17 ^a^	250.78 ± 8.52 ^a^
Kaempferol-3-O-acetyl-glucoside	44.40 ± 4.59 ^b^	64.90 ± 2.86 ^a^	52.69 ± 3.07 ^b^	37.44 ± 1.47 ^c^	72.09 ± 10.70 ^a^	54.23 ± 4.44 ^b^	68.20 ± 9.04 ^a^	69.39 ± 4.66 ^a^
Isorhamnetin pentoside	58.44 ± 3.13 ^a^	34.21 ± 3.21 ^b^	36.85 ± 3.33 ^b^	35.53 ± 2.44 ^b^	56.70 ± 6.02 ^a^	54.84 ± 3.01 ^a^	33.14 ± 0.62 ^b^	31.87 ± 1.82 ^b^
Afzelin	614.96 ± 21.00 ^b^	647.37 ± 8.44 ^a^	504.66 ± 4.65 ^d^	454.80 ± 17.63 ^e^	663.35 ± 9.73 ^a^	536.83 ± 28.39 ^c^	623.99 ± 24.36 ^a^	632.09 ± 18.39 ^a^
Quercetin-3-O-acetyl-rhamnoside	328.08 ± 24.19 ^a^	361.05 ± 33.00 ^a^	113.19 ± 5.91 ^e^	278.44 ± 19.89 ^c^	316.47 ± 25.59 ^b^	309.57 ± 14.17 ^b^	326.16 ± 13.12 ^a^	243.87 ± 9.14 ^d^
Kaempferol-3-O-(6-acetyl-galactoside)-7-O-rhamnoside	66.61 ± 3.48 ^e^	129.69 ± 7.93 ^b^	36.79 ± 7.41 ^f^	106.59 ± 6.28 ^c^	73.17 ± 2.50 ^d^	59.81 ± 3.60 ^e^	140.78 ± 4.25 ^a^	144.09 ± 8.25 ^a^
Flavon-3-ols								
(−)-epicatechin	1182.07 ± 38.48 ^d^	1994.77 ± 60.04 ^a^	1045.85 ± 32.83 ^e^	1053.99 ± 24.34 ^e^	1191.01 ± 78.38 ^d^	1384.90 ± 48.99 ^c^	1692.80 ± 32.04 ^b^	1903.47 ± 79.09 ^a^

Values were expressed as mean ± standard deviation (SD) (n = 3); different superscript letters within the same row indicate statistically significant differences according to Duncan’s least significant difference (LSD) procedure at a 5% significance level.

**Table 2:** The list of *A. spicata* leaf sites with their administrative units, geographical coordinates, and habitat type.

Site Number	Site Name	Administrative Unit	Habitat Type	Longitude (°E)	Latitude (°N)	Altitude m
1	Bartlaukis	Telšiai distr.	Outskirts	55.695507	22.388133	252.0
2	Kęsai	Telšiai distr.	Outskirts	55.689364	22.47016	208.0
3.	Nevardėnai	Telšiai distr.	Outskirts	55.685624	22.442243	207.0
4.	Klaišiškiai	Telšiai distr.	Outskirts	55.690565	22.408210	167.0
5.	Kaunas	Kaunas city	Outskirts	54.898521	23.903597	47.0
6.	Vievis	Elektrėnai distr.	Forest	54.765872	24.823586	112.2
7.	Marcinkonys	Varėna distr.	Forest	54.061529	24.398346	137.0
8.	Darželiai	Varėna distr.	Forest	54.022878	24.332646	118.9

## Data Availability

The data presented in this study is available in the article.

## Supplementary Materials

The following supporting information can be downloaded at: https://www.mdpi.com/article/10.3390/plants14020221/s1, Table S1. Average air temperatures (°C) during the vegetation period (April–September 2023) in the areas of investigated populations of *Amelanchier × spicata* (Lam.) K. Koch; Table S2. The amounts of precipitation (mm) during the vegetation period (April–September 2023) in the areas of investigated populations of *Amelanchier × spicata* (Lam.) K. Koch; Table S3. The soil pH and soil type in the areas of investigated populations of *Amelanchier × spicata* (Lam.) K. Koch; Table S4. HPLC-MS/PDA data of *Amelanchier × spicata* (Lam.) K. Koch leaf samples.
