# Supplementary material for: Comparative Analysis of Phenolic Profiles and Antioxidant Activity in the Leaves of Invasive Amelanchier × spicata (Lam.) K. Koch in Lithuania
Source: Plants (Basel). 2025 Jan 14;14(2):221. doi: 10.3390/plants14020221 (PMC11769043; doi:10.3390/plants14020221)
Supplement: Supplementary file 1 [file plants-14-00221-s001.zip › plants-3379596-supplementary.pdf]

Table S1. Average air temperatures (°C) during the vegetation period (April – September, 2023) in the areas of investigated populations of *Amelanchier* × *spicata* (Lam.) K. Koch.

Population number	Habitat name and region	April	May	June	July	August	September
1	Bartlaukis (Telšiai district)	7.5	12.3	13.1	16.8	18.3	16.2
2	Kęsai (Telšiai district)	7.5	12.3	13.1	16.8	18.3	16.2
3	Nevardėnai (Telšiai district)	7.5	12.3	13.1	16.8	18.3	16.2
4	Klaišiškiai (Telšiai district)	7.5	12.3	13.1	16.8	18.3	16.2
5	Kaunas (Kaunas manucipality)	8.4	12.7	17.3	17.9	20.1	17.1
6	Vievis (Elektrėnai district)	8.6	13.1	17.5	18.3	20.5	17.4
7	Marcinkonys (Varėna district)	8.2	11.9	17.0	17.8	19.8	15.9
8	Darželiai (Varėna district)	8.2	11.9	17.0	17.8	19.8	15.9

Table S2. The amounts of precipitation (mm) during the vegetation period (April – September, 2023) in the areas of investigated populations of *Amelanchier* × *spicata* (Lam.) K. Koch.

Population number	Habitat name and region	April	May	June	July	August	September
1	Bartlaukis (Telšiai district)	11.8	10.1	21.2	62.0	155.1	51.8
2	Kęsai (Telšiai district)	11.8	10.1	21.2	62.0	155.1	51.8
3	Nevardėnai (Telšiai district)	11.8	10.1	21.2	62.0	155.1	51.8
4	Klaišiškiai (Telšiai district)	11.8	10.1	21.2	62.0	155.1	51.8
5	Kaunas (Kaunas manucipality)	26.7	14.3	64.0	36.8	96.2	11.6
6	Vievis (Elektrėnai district)	30.8	21.2	37.5	32.1	91.3	15.3
7	Marcinkonys (Varėna district)	49.2	30.6	87.3	44.0	129.5	19.6
8	Darželiai (Varėna district)	49.2	30.6	87.3	44.0	129.5	19.6

Table S3. The soil pH and soil type in the areas of investigated populations of *Amelanchier* × *spicata* (Lam.) K. Koch.

Population number	Habitat name and region	Soil pH	Soil type
1	Bartlaukis (Telšiai district)	4.5–5.0	Glacial till
2	Kęsai (Telšiai district)	4.5–5.0	Glacial till
3	Nevardėnai (Telšiai district)	4.5–5.0	Glacial till
4	Klaišiškiai (Telšiai district)	4.5–5.0	Glacial till
5	Kaunas (Kaunas manucipality)	< 6.1	Periglacial till
6	Vievis (Elektrėnai district)	< 4.5	Glaciofluvial sand
7	Marcinkonys (Varėna district)	< 4.5	Glaciofluvial sand
8	Darželiai (Varėna district)	< 4.5	Glaciofluvial sand

Table S4. HPLC-MS/PDA data of *Amelanchier* × *spicata* (Lam.) K. Koch leaf samples.

No	Compound	UV λmax	[M-H]- (m/z)	References
1	Neochlorogenic acid*	325	353	[96,97,98]
2	Chlorogenic acid*	325	353	[19]
3	4-O-caffeoylquinic acid *		353	[99]
4	Caffeoylquinic acid derivative**	317	191, 353	[99]
5	Hydroxycinnamic acid derivative**	327	297, 355	[19,100]
6	Coumaric acid*	226, 294	173, 191, 337	[101]
7	4-p-coumaroylquinic acid*	311	173, 191, 337	[99]
8	1,5-dicaffeoylquinic acid*	327	335, 515	[99]
9	Protocatechuic acid*	258, 293	153	[18,25]
10	Quercetin 3-arabinoside 7-glucoside**	256, 354	301, 595	[21]
11	Rutin*	255, 353	301, 609	[25]
12	Kaempferol 3-sambubioside **	265, 353	285, 579	[21,25]
13	Quercetin 3-O-robinobioside**	256, 354	609	[25,19]
14	Hyperoside *	255, 354	463	[100]
15	Isoquercitrin *	255, 353	463	[100]
16	Isorhamnetin-3-rutinoside*	254, 353	315, 623	[102]
17	Kaempferol-3-rutinoside*	265, 347	285, 593	[102]
18	Isorhamnetin derivative**	254, 353	315, 623	[102]
19	Reynoutrin*	255, 353	433	[100]
20	Kaempferol derivative**	266, 348	285, 451	[100]
21	Astragalin *	265,346	285, 447	[100]
22	Quercetin 3-O-malonylglucoside*	255, 354	301, 549	[25]
23	Quercetin 3-O-α-L-arabinopyranoside**	255, 352	301, 433	[101]
24	Isorhamnetin 3-O-glucoside*	254, 353	315, 477, 549	[103]

Table continued

25	Quercitrin*		301, 447	[100]
26	Kaempferol 3-O-arabinoside*	265, 342	285, 417, 625	[104]
27	Kaempferol 3-O-acetyl-glucoside**	265, 347	285, 489, 604	[25,102]
28	Isorhamnetin pentoside**	253, 353	315, 447	[105]
29	Afzelin*	265, 347	285, 431	[103]
30	Quercetin 3-O-acetyl-rhamnoside**	255, 347	301, 489	[101]
31	Kaempferol 3-O-(6"-acetyl-galactoside) 7-O-rhamnoside**	264, 342	285, 635	[103]
32	(-)-Epicatechin*	280	289	[18,104]

*identification of these compounds made by comparison with standards

**were tentatively quantified using calibration curves of substances with similar chemical structure
